# The “Egg of Columbus” for Making the World's Toughest Fibres

**DOI:** 10.1371/journal.pone.0093079

**Published:** 2014-04-02

**Authors:** Nicola M. Pugno

**Affiliations:** 1 Laboratory of Bio-Inspired & Graphene Nanomechanics, Department of Civil, Environmental and Mechanical Engineering, Università di Trento, Trento, Italy; 2 Center for Materials and Microsystems, Fondazione Bruno Kessler, Povo (Trento), Italy; 3 School of Engineering & Materials Science, Queen Mary University of London, London, United Kingdom; Massachusetts Institute of Technology, United States of America

## Abstract

In this letter we present the “Egg of Columbus” for making fibres with unprecedented toughness: a slider, in the simplest form just a knot, is introduced as frictional element to dissipate additional energy and thus demonstrating the existence of a previously “hidden” toughness. The proof of concept is experimentally realized making the world's toughest fibre, increasing the toughness modulus of a commercial Endumax macroscopic fibre from 44 J/g up to 1070 J/g (and of a zylon microfiber from 20 J/g up to 1400 J/g). The ideal upperbound toughness is expected for graphene, with a theoretical value of ∼10^5^ J/g. This new concept, able of maximizing (one fold increment) the structural robustness, could explain the mysterious abundance of knot formations, in spite of their incremental energy cost and topological difficulty, in biological evolved structures, such as DNA strands and proteins.

## Introduction

A great flourish of interest in the development of new high-strength and high-toughness materials is taking place in contemporary materials science, with the aim of surpassing the mechanical properties of commercial high-performance fibres. Recently, macroscopic buckypapers [1]–[5], nanotube bundles [5]–[13] and graphene sheets [14]–[18] have been manufactured. While their macroscopic strength remains 1–2 orders of magnitude lower than their theoretical strength, and is thus comparable to that of current commercial fibres, recent progress has been made in significantly increasing toughness. In particular, researchers have produced extremely tough nanotube fibres with toughness modulus values of up to 570 J/g [8], 870 J/g [13] and very recently, including graphene, reaching 970 J/g [8], thus well surpassing that of Kevlar (∼80 J/g [8]) and even spider silk (∼170 J/g [8], with a record for a giant riverine orb spider of ∼390 J/g [19]). In this letter, thanks to a new paradigm based on structural mechanics rather than on materials science, we present the “Egg of Columbus” for making fibres with unprecedented toughness: a slider, in the simplest form just a knot, is introduced as frictional element to dissipate energy and in general to reshape the fibre constitutive law, showing evidence of a previously “hidden” toughness, strictly related to the specific strength of the material. The result is a nearly perfectly plastic constitutive law, with a shape mimicking that of spider silk. The proof of concept is experimentally realized making the world's toughest fibre, increasing the toughness modulus of a commercial Endumax macroscopic fibre from 44 J/g up to 1070 J/g (and of a zylon microfiber from 20 J/g up to 1400 J/g). This new concept, able of maximizing the structural robustness, could explain the mysterious abundance of knot formations, in spite of their incremental energy cost and topological difficulty, in biological evolved structures, such as DNA strands and proteins [20]–[30]. The ideal upperbound toughness is expected for graphene or carbon nanotubes [31]–[35], with a theoretical value of ∼10^5^ J/g.

## The Concept

**Figure 1:**
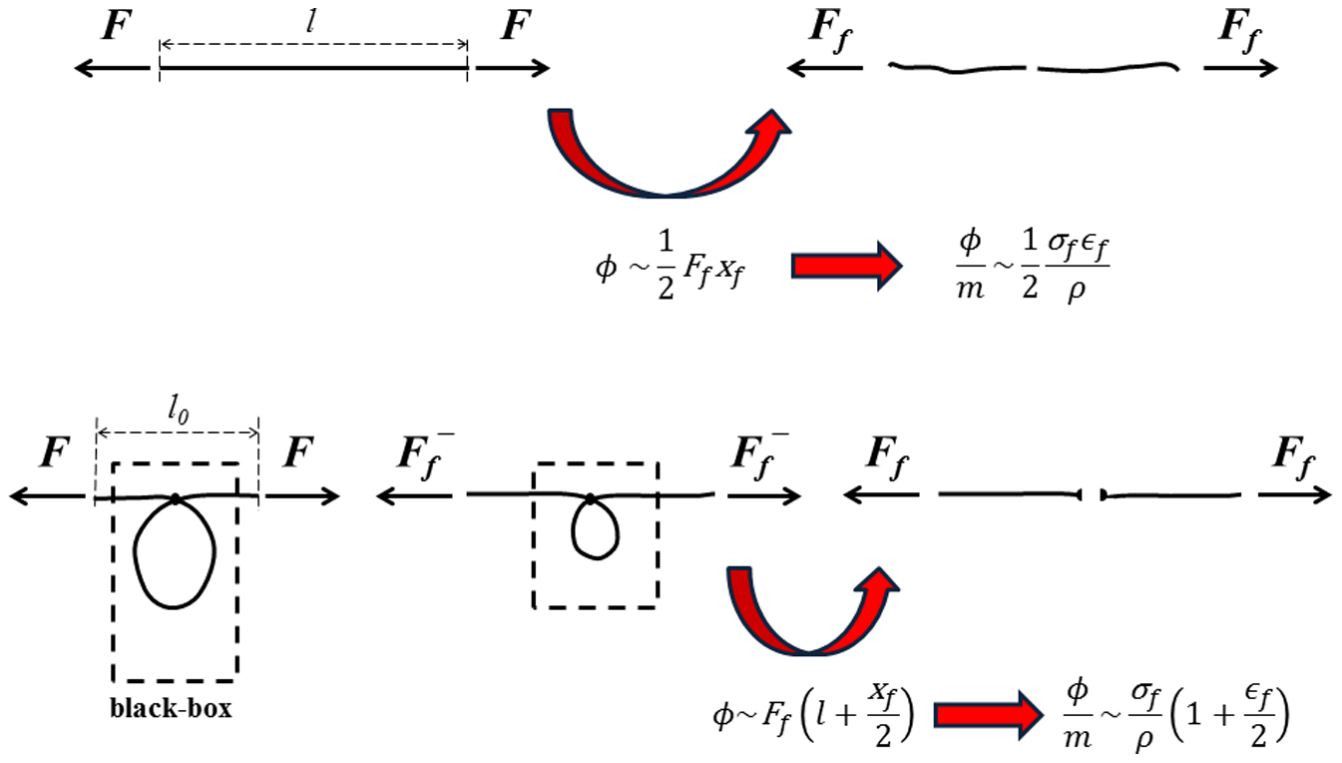
Concept. The classical fibre dissipates during fracture its cumulated strain energy, thus displaying a toughness modulus of 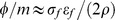 (the factor 2 must be replaced by an other number for nonlinear elastic fibres). In contrast, a fibre with a slider, e.g. knot, can dissipate much more energy, thanks to a sliding friction force. The upper limit of the toughness in this case is constituted by the product of a force 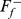 just below the breaking force 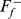 and a displacement equal to the entire fibre length l, thus reaching a toughness modulus of 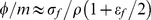. Accordingly, a huge (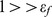) previously “hidden” toughness 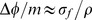 naturally emerges.

The *concept* is thus based on the introduction of appropriate sliders, even simple knots, as frictional elements for energy dissipation in one dimensional elements such as fibres. Part of the energy is dissipated through friction by the fibre sliding in the slider along an extra-length of the fibre, e.g. a loop, additionally to the intrinsic stretching energy dissipated by fracture and kinetic energy when the fibre breaks. The concept is summarized in Fig. 1.

**Figure 2:**
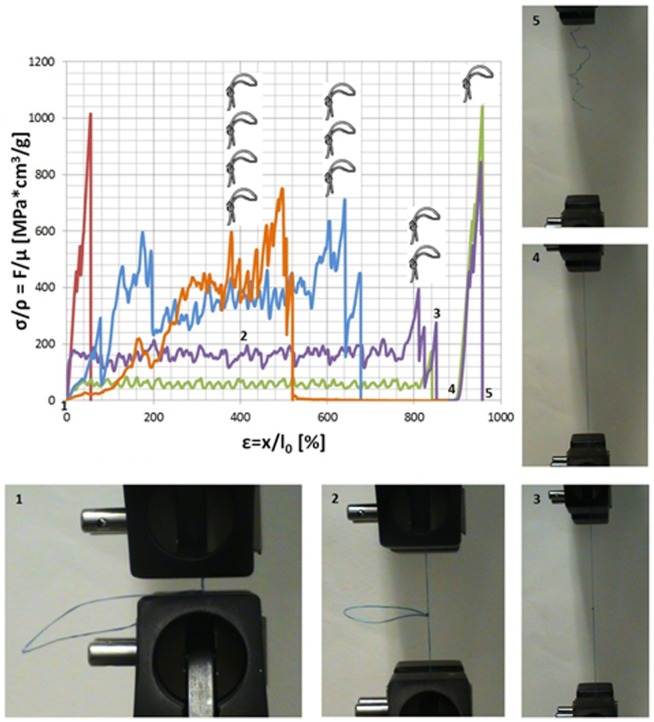
Proof of concept. Specific force-displacement or stress-strain curves of knotted and unknotted Dyneema fibres (the number of knots is depicted for each curve; 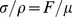 with 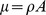 mass per unit length, given in MPa*cm^3^/g or, equivalently, in J/g; test parameters are dx/dt = 2 mm/min, l_0_ = 10 mm, l = 100 mm, μ = 0.0361 g/m). The appearance with the knot of the hidden toughness, the plastic-like plateau absent in the constitutive law of the unknotted fibre (27 J/g), is evident. For 1 and 2 coils the knot unties (peculiar mechanism) and stress goes to zero, then the fibres extends, deforms and fractures at the pristine fibre strength, with an increment in the toughness of up to 722% (2 coils, 195 J/g). For 3 coils the dissipated energy is further increased up to a maximal value of 320 J/g, corresponding to a toughness increment of 1185%. For 4 coils, premature failure leads to a reduction in both toughness and failure strain. The total hidden toughness is given by the specific strength, thus for this fibre it is close to 1000 J/g.

**Figure 3:**
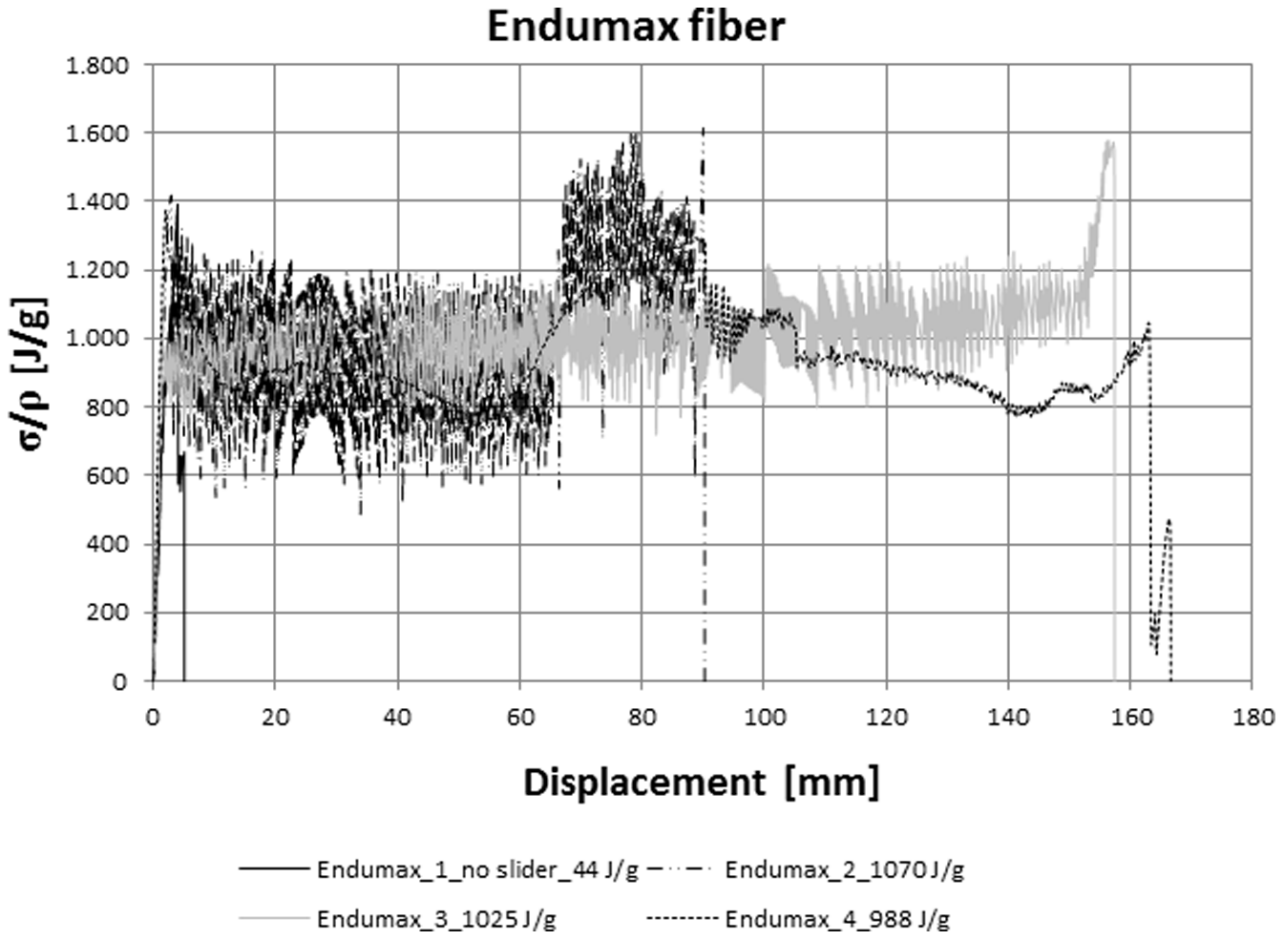
World toughness record (work in progress, with a zylon microfiber we have already achieved a toughness modulus of 1400 J/g starting from its intrinsic value of 20 J/g - preliminary results reported in the ERC proposal “Knotough”). Force vs displacement curve of Endumax fibres with (samples 2,3,4) or without a slider (sample 4). The pristine fibre has a toughness modulus of 44 J/g. The introduction of the slider dramatically changes the scenario: a long plastic-like plateau clearly emerges thanks the presence of the slider and allows the dissipation of a huge amount of energy, approaching an unprecedented toughness modulus of 1070 J/g (other two tests, leading to 988 and 1025 J/g are shown). The specific strength and thus maximal achievable toughness is for this fibre of about 1600 J/g (see stress peak in the figure).

In particular, the energy at break 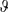 per unit mass 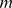 of a fibre having cross-sectional area *A*, length *l*, Young's modulus *E*, strength 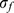 and mass density 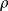, can be calculated from the load-displacement curve as 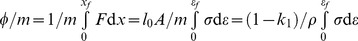, where *F* is the force, *x* is the displacement, 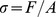 is the stress, 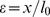 is the apparent strain, 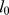 is the end-to-end length of the fibre and 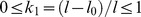 is a slider parameter whereas the subscript *f* denotes final values, see Fig. 1. Accordingly 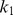 represents the ratio between the extra-length, e.g. of the loop, and the total length of the fibre. For a linear elastic fibre, classical thus without an extra-length or slider (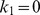), this simply yields 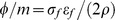, where 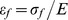; in contrast, for “knotted fibres” the situation changes dramatically and the toughness increases enormously (see expression of 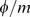, where 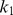 appears in both the constant of proportionality and in the definition of the strain and thus cannot be directly evinced from the stress-strain curve). For the sake of simplicity, let us consider a fibre forming a large slip-loop in a slider, e.g. a knot, with the two clamped fibre ends initially fixed as close as possible to the slider, Fig. 1. When a knot is inserted in a fiber, the fiber strength in general decreases due to the stress concentration imposed by the presence of the knot; the related “knot strength” of the fibre is here denoted by 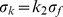, where accordingly 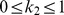. Considering an independent slider/knot can mitigate this restriction. During fibre tension, first the strain increases with the fibre sliding through the slider at a mean stress plateau value of 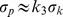, where 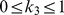 denotes the ratio between plateau stress and slider/knot fibre strength. This sliding phase takes place for a displacement 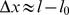, ideally at a force just below the breaking force 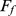 of the fibre, in order to maximize the dissipated energy; then, the slip-loop tightens (it could also unfasten, depending on the type of slider/knot topology, e.g. see Fig. 2) and the fibre deforms and finally breaks. Thus, in the stress-strain curve a long plastic-like plateau naturally emerges (Figs. 2 and 3) thanks to the presence of the slider. The increment of the final strain is 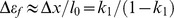 and can be precisely tuned selecting an appropriate value of 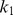. The strength of the fibre with the slider (e.g. “knot strength”) should ideally approach that of the pristine fibre in order to minimize the negative strength variation 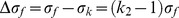. Accordingly, the increment in toughness modulus, or previously “hidden toughness”, is huge and given by 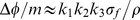 approaching the enormous value of 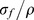, which is the fibre specific strength. Regarding the estimation of the Young's modulus 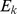 of the knotted fibre, we simply (series of compliances) find 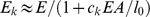, where 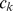 is the knot compliance and thus a weak softening 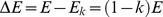 (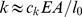 for 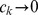, i.e. tight knots, for which 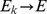) is expected. More in general, the application of this concept allow to reshape the constitutive law of the fibre in terms of variations of the four main engineering properties 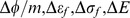, i.e. toughness modulus, failure strain, strength and Young's modulus, respectively. In particular, we can mimic the constitutive law of spider silk, crucial for structural robustness [31], with the friction in the slider that plays the role of the hydrogen bond breaking in the silk, which is responsible for its dissipative plastic-like plateau. Moreover, the usually competing material properties of strength and toughness (we are considering here the toughness modulus rather than so-called fracture toughness) are reconciled: high strength and simultaneously super-tough fibres become feasible, of course at the expense of a larger elongation, the latter being either positive or negative depending on the specific application. Note, that this mechanism leads to one-shot plasticity, as the real plasticity does. Of course changing the application points of the forces, in order to make cycles of the extra-length of the fiber (i.e. extending and reducing the length of the loop) can lead to systems with multiple-shots plasticity. Also note that, especially in this last case, there may be some associated effects with the optimal conditions 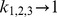 that may limit the maximum theoretical toughness. For example, if the plateau stress is very high the friction of the fiber through the knot is very high, which may result in serious degradation of the fiber as it slides through the knot especially for multiple cycles. This degradation may lead to premature failure that may be well under the original strength of the fiber. Also, high values of the plateau stress will most likely restrict the value of the failure stress of the knotted fiber. Thus 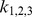 are not fully independent and are evolving parameters. It is clear that in order to maximize the dissipated energy and therefore the toughness modulus, the specific strength of the fibre must be high, i.e., 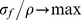 (fibre condition n. 0), and the following 3 conditions must be met: the sliding length must be maximized (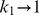, geometrical condition n. 1); the fibre must display minimal fragilization due to the presence of the slider (expected as a consequence of the applied additional stresses; 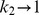, fibre-slider condition n. 2); the slider must ensure an initial stress plateau as flat and as high as possible (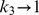, slider condition n. 3). Clearly, the mass of the slider should also be minimized, but its influence on the toughness modulus approaches zero for increasing sliding/fiber length. These 1+3 main conditions can substantially be achieved through careful choice of fibres and sliders. For example hundreds of knots, widely used in climbing, sailing and fishing activities, are currently known, and multiple knots can be realized too, allowing the design of complex, e.g. multi-plateau, constitutive laws and architectures, e.g. ropes, fabrics, hierarchical reinforcements for composites, etc.

Moreover, note that this concept partially applies also to other systems not in tension and without knots, e.g. curved wires under bending or torsion in addition to stretching: the wire will exhibit an increasing stiffness with deformation and the resulting toughness will be much superior than that of the related initially straight wire; eventually, the wire will straighten and fracture at the same load of an initially straight wire; however note that, in this case, the force is not maximal from the beginning and thus the gain in toughness is not maximized, as for the case of fibres working just below the fracture force from the beginning. Formation of plastic hinges could be desired as observed in tough helical carbon nanotubes [34]–[35] or in contrast could be a limitation for energy storage and thus super-elastic, e.g. NiTi, alloys, could also be good candidates.

## The Proof of Concept

The *proof of concept* is realized considering commercial Dyneema fibres (fishing line, multifilament composed by 200 filaments), which combine a high-strength with low density, i.e. quite high specific strength (and stiffness). We tested them in a uniaxial loading tensile testing machine (MTS, Insight). The measured constitute law of this fibre is reported in Fig. 2 (curve without the symbol of knot) and shows a specific strength of 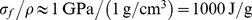 and a toughness modulus of 27 J/g. Note that the value of the specific strength, which according to our previous considerations also denotes the hidden toughness, suggests a huge margin for increasing the toughness of this fibre. Additionally, these fibres display reduced fragilization when tied into slip-knots, allowing us to prove the concept with a simple double knot, Fig. 2, that however is itself sliding along the fibre and is not an independent slider and thus does not allow us to reach a high stress plateau: here we have deliberately not optimized the knot in order to prove the robustness of the concept (in our cases 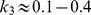, depending on the number of coils, and 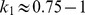; these numbers are roughly estimated by their definitions from the experimental force vs. displacements curves). We have considered also different strain rates (concluding the tensile tests in characteristic times of seconds) with the aim of reducing the stick-slips in the plateau but without any significant improvement.

The adopted knots were simple, double, triple or quadruple overhand knots (double topology reported in Fig. 2). Different numbers of coils (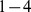, specified for each curve) are considered in order to maximize the friction force during sliding without leading to premature fibre fracture before full knot tightening. The fibre length is *l* = 10 cm whereas the selected initial value of the end-to-end length was fixed at 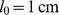, thus 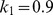 and 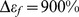. Note that the strain is here fictitious –due to the arbitrary choice of the initial length– whereas the displacement is the physical quantity. The measured specific stress-strain curves of knotted fibres are shown in Fig. 2 and compared to that of the unknotted fibre. The expected appearance with the knot of a previously hidden toughness, and thus of a, even if here irregular, plastic-like plateau, absent in the constitutive law of the unknotted fibre, is evident. For 1 and 2 coils a peculiar mechanism is observed, leading to the optimal condition 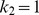 and no strength reduction, i.e. 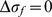: the knot unfastens after the full closure of the loop (this is the reason for which the stress goes to zero in the related curves), then the fibre extends, deforms and fractures at exactly the pristine fibre strength. For 1 coil the toughness modulus reaches 88 J/g whereas for 2 coils a value of 195 J/g is obtained, thanks to an increment in the toughness up to 722%, without any strength reduction. The constitutive law of this fibre resembles that of spider silk (in terms of both toughness modulus and strength). For 3 coils the dissipated energy further increases up to a value of 320 J/g, corresponding to a toughness increment of 1185%, with a strength reduction of ∼25%. For 4 coils premature failure leads to a reduction in both failure strain and toughness modulus, of 148 J/g, with a similar strength reduction. We conclude that a number of 3 coils maximizes the toughness of our first specific knot-fibre system. These set of experiments prove the robustness of the concept.

We finally consider independent sliders/knots and Endumax fibres. Following the previous procedure a more regular sliding, thanks to the independency of the slider that thus remains fixed during the fibre sliding (specific details of the slider are under patenting) is achieved and results in a more regular plastic-like plateau, see Fig. 3. The initial toughness modulus of the pristine fibre thus without slider is 44 J/g. With the introduction of the slider we obtained 988, 1025 and finally 1070 J/g, thus surpassing the unprecedented value of 1000 J/g. Further improvements are expected for this specific fibre, up to a toughness modulus close to the fibre specific strength that we have measured as 1600 J/g (see stress peak in Fig. 3); with a zylon microfiber we have already achieved a toughness modulus of 1400 J/g starting from its intrinsic value of 20 J/g (preliminary results reported in the ERC proposal “Knotough”).

## Towards the Solution of the Mystery of Slipknots in Biology

This new concept could have important consequences in Biology. In particular, proteins separated by a billion years of evolution often display remarkable similarities and functions. Slipknots, for example, have been found in proteins as well as in DNA strands [20]–[30] and are conserved in different families and species. This happens even if the folding process resulting in the formation of knots is intrinsically more energetically expensive and topologically difficult than the process of producing unknotted proteins. Thus knotting might seem unlikely to occur during evolution but, in contrast and still surprising, it does regularly occur. Specifically, Sulkowska el al. [21]–[23] have quantified how knots and slipknots, instead of being discarded through the process of evolution, are strongly conserved in proteins. This confirms that, despite the larger energy cost and topological difficulty in the formation of knots, they are somehow advantageous and important to the function of the protein. Moreover, according to the same authors, knots and slipknots take place at specific points of larger flexibility and contribute to the stability of the location of the protein, e.g. in the membrane barriers of cells. However, the significant extra effort to fold into knotted shapes must have a biological payoff or Nature would have selected a less expensive path. The stability may not be sufficient to justify alone this payoff. According to the concept that we have presented in this paper we here suggest a new plausible important reason for the appearance of slipknots in Biology: biological structures may have evolved with knots in order to easily but dramatically (one fold) increase their robustness. This huge robustness enhancement of the protein could be crucial for resisting against different types of diseases or viceversa to design new types of more robust proteins for disease treatments.

Also coiled structures are examples of other preferred solutions in Nature, e.g. the topology of the primary and secondary structure of the DNA, the coiled coil structural motif of proteins (in which alpha-helices are coiled together like the strands of a rope; dimers and trimers are the most common types); and as previously discussed, also coiled structures –in addition to knots– may lead to a higher robustness thanks to a larger ultimate displacement.

## Conclusions

Summarizing, thanks to the new evidence of existence of a previously “hidden” toughness, much higher toughness levels, which can surpass the highest-known values in the literature, could be obtained in the near future. We can apply our concept to current high-strength or high-tough fibres, e.g. graphene/nanotube-based (which have also shown to be resilient to knots [32]). Note that, considering the theoretical nanotube/graphene strength, an ideal upperbound toughness theoretical limit of about 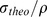
∼10^5^ J/g is computed; moreover, since smaller is stronger, the hidden toughness can be more easily observed reducing the system size-scale. Even spiders can thus do better. For example, the giant riverine orb spider [19] seems to have optimized toughness during the last ∼200 millions of years of evolution; in spite of this, for the mentioned spider we compute a toughness limit of 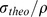
∼1380 J/g against its measured value and actual record of ∼390 J/g (we have assumed a mean density of ∼1.34 g/cm^3^). Thus, even this spider (or us, using its silk and our concept) could further improve the toughness of its silk, by a factor of ∼350%. During the next millions of years this percentage of hidden toughness will be probably further reduced by evolution and the same appearance of knots in spider silks and webs cannot be excluded, as already observed in proteins and DNA strands. This “egg of Columbus” could thus be used by evolution for maximizing (one fold increment) the robustness of biological systems and for suggesting us new strategies to design innovative types of more robust proteins for disease treatments or to design unprecedented super-tough materials.
